# Biophysical Characterization and Activity of Lymphostatin, a Multifunctional Virulence Factor of Attaching and Effacing *Escherichia coli* *

**DOI:** 10.1074/jbc.M115.709600

**Published:** 2016-01-19

**Authors:** Robin L. Cassady-Cain, Elizabeth A. Blackburn, Husam Alsarraf, Emil Dedic, Andrew G. Bease, Bettina Böttcher, René Jørgensen, Martin Wear, Mark P. Stevens

**Affiliations:** From the ‡ Roslin Institute, Roslin Institute and Royal (Dick) School of Veterinary Studies, University of Edinburgh, Easter Bush, Midlothian EH25 9RG, United Kingdom,; the § Centre for Translational and Chemical Biology (CTCB), University of Edinburgh, Michael Swann Building, King's Buildings, Edinburgh EH9 3BF, United Kingdom, and; the ¶ Department of Microbiology & Infection Control, Statens Serum Institut, Artillerivej 5, DK-2300 Copenhagen S, Denmark

**Keywords:** cell proliferation, circular dichroism (CD), electron microscopy (EM), Escherichia coli (E. coli), glycosyltransferase, small-angle X-ray scattering (SAXS), structural biology, T-cell, LifA, lymphostatin

## Abstract

Attaching and effacing *Escherichia coli* cause diarrhea and typically produce lymphostatin (LifA), an inhibitor of mitogen-activated proliferation of lymphocytes and pro-inflammatory cytokine synthesis. A near-identical factor (Efa1) has been reported to mediate adherence of *E. coli* to epithelial cells. An amino-terminal region of LifA shares homology with the catalytic domain of the large clostridial toxins, which are retaining glycosyltransferases with a D*X*D motif involved in binding of a metal ion. Understanding the mode(s) of action of lymphostatin has been constrained by difficulties obtaining a stably transformed plasmid expression clone. We constructed a tightly inducible clone of enteropathogenic *E. coli* O127:H6 *lifA* for affinity purification of lymphostatin. The purified protein inhibited mitogen-activated proliferation of bovine T lymphocytes in the femtomolar range. It is a monomer in solution and the molecular envelope was determined using both transmission electron microscopy and small-angle x-ray scattering. Domain architecture was further studied by limited proteolysis. The largest proteolytic fragment containing the putative glycosyltransferase domain was tested in isolation for activity against T cells, and was not sufficient for activity. Tryptophan fluorescence studies indicated thatlymphostatin binds uridine diphosphate-*N*-acetylglucosamine (UDP-GlcNAc) but not UDP-glucose (UDP-Glc). Substitution of the predicted D*X*D glycosyltransferase motif with alanine residues abolished UDP-GlcNAc binding and lymphostatin activity, although other biophysical properties were unchanged. The data indicate that lymphostatin has UDP-sugar binding potential that is critical for activity, and is a major leap toward identifying the nature and consequences of modifications of host cell factors.

## Introduction

Enterohemorrhagic *Escherichia coli* (EHEC)^4^ and enteropathogenic *E. coli* (EPEC) are enteric bacterial pathogens of worldwide importance. Both pathotypes colonize intestinal mucosa via the formation of “attaching and effacing” lesions in a manner that requires a Type III protein secretion system, as well as accessory virulence factors including surface adhesins (1). Lymphostatin (also known as LifA) is a chromosomally encoded protein with a predicted molecular mass of 365 kDa produced by most EPEC and non-O157 EHEC strains (2). We have previously demonstrated that lymphostatin is required for intestinal colonization of calves by non-O157 EHEC serogroups O5, O111 (3), and O26 (4), and it also promotes colonization of the murine intestines and colonic hyperplasia by the attaching and effacing pathogen *Citrobacter rodentium* (5). *Chlamydia* species also contain a family of proteins that have homology to lymphostatin, and which have been implied to act as cytotoxins (6).

Lymphostatin was first described as the factor required for inhibition of mitogen-activated proliferation of lymphocytes by enteropathogenic *E. coli* O127:H6 lysates (2). This activity has been demonstrated against lymphocytes from peripheral blood and the intestines (3, 7) and is not associated with direct cytotoxicity. Peripheral blood mononuclear cells from mice, cattle, and humans are sensitive to lymphostatin (2, 3, 5). Lymphostatin has also been reported to inhibit the production of pro-inflammatory cytokines including IL-2, -4, -5, and interferon-γ (7), and it has been suggested that it may therefore interfere with the induction of innate and adaptive immune responses. In the same year as LifA was described in EPEC, a near identical factor was described in EHEC O111:H− that was associated with bacterial adherence to cultured epithelial cells. The authors named the factor EHEC factor for adherence 1 (Efa1), however, it has 97.4% amino acid identity to lymphostatin, and they are likely equivalent proteins (8). Although a direct role of Efa1 in adherence has been reported using rabbit EPEC (9), mutations in some strains impair expression and secretion of Type III secreted proteins required for attaching and effacing-lesion formation (3). Furthermore, it has recently also been reported that lymphostatin can be secreted via the type III secretion system, but its functions once injected into host cells are unknown (10).

Understanding of the mode of action of lymphostatin has been constrained by the instability of plasmid clones and difficulties in obtaining full-length purified protein (2). Furthermore, even plasmid-driven soluble expression of smaller fragments of lymphostatin has proven to be difficult (11). Bioinformatic analysis has identified homology between the amino-terminal of LifA/Efa1 and the catalytic glycosyltransferase domain of the LCTs (2, 8). These clostridial cytotoxic molecules are large proteins whose catalytic domain glycosylates Rho-family GTPases that regulate the actin network (12). They are retaining enzymes with a GT-A fold, which belong to glycosyltransferase family 44 and are characterized by having a D*X*D (Asp-Xaa-Asp) motif involved in metal ion binding together with the diphosphate moiety of the UDP-sugar donor (13). It is a multistep reaction dependent on the D*X*D motif, where sugar binding and hydrolysis is followed by transfer of the released sugar to an acceptor protein (reviewed in Ref. 12).

In addition, recent studies show that attaching and effacing *E. coli* express another novel protein, NleB, which is an effector glycosyltransferase injected into host cells upon infection. NleB blocks death receptor-induced apoptosis and promotes intestinal colonization (14, 15), as part of a suite of *E. coli* effectors that influence NF-κB signaling in mammalian cells (reviewed in Ref. 16). NleB uses uridine diphosphate *N*-acetylglucosamine (UDP-GlcNAc) as its donor sugar and covalently *N*-links a GlcNAc to a conserved arginine residue in death domain containing adaptor proteins (14, 15). This modification had not previously been described and most GlcNAc addition to proteins occurs via *O-*linkage on serine/threonine residues (14, 15). Apart from the glycosyltransferase domain, searches also identified a *Yersinia* YopT-like cysteine protease (CP) motif in the sequence of lymphostatin (17). These features represent a small portion of the primary sequence of lymphostatin, and are restricted to the N-terminal third of the protein. Although one report claimed that deletion of the predicted glycosyltransferase and cysteine protease motifs attenuated *C. rodentium* in mice (5), close inspection reveals that stop codons were introduced that resulted in protein truncation at the deletion site rather than in-frame mutations, making the results difficult to interpret (4). Given its large size and the paucity in understanding how lymphostatin is able to carry out its activities, we sought to produce a full-length recombinant lymphostatin and characterize its structural and biophysical features, as well as its effects on activated T cells. Here we show that full-length lymphostatin can be expressed as a folded, biologically active recombinant protein that inhibits bovine T cell proliferation at very low concentrations. Furthermore, like other glycosyltransferases, it has sugar-binding potential, and both its biological activity and sugar binding is dependent on a D*X*D motif, which has been implicated in the activity of glycosyltransferases. Although the activity of lymphostatin was identified more than a decade ago, this is the first significant step forward in understanding the mechanisms underlying its intrinsic activities.

## Experimental Procedures

### 

#### 

##### Cloning

The full-length gene encoding lymphostatin (∼9.6 kb) was cloned using the commercially available Expresso Rhamnose cloning and expression system (Lucigen Inc.) from the prototype enteropathogenic *E. coli* serotype O127:H6 strain E2348/69. The lymphostatin gene was amplified using genomic DNA from E2348/69 as lymphostatin activity was first described in this strain (2). The pRham vector incorporates a C-terminal in-frame His_6_ tag. Amplicons were generated using the primers EXPLifA_FOR, 5′-GAAGGAGATATACATATGAGACTGCCAGAGAAAGTTCTT-3′ and EXPLifA_REV, 5′-GTGATGGTGGTGATGATGGTTAAAAAGGTTGTCACCATT-3′ with PHUSION proofreading polymerase (Thermo Scientific). Amplicons of the appropriate size were isolated by agarose gel electrophoresis and purified using Geneclean II (MP Biomedicals, Inc.). The amplicons were cloned into pre-linearized pRham vector by homologous recombination in *E. cloni 10G*® (Lucigen Inc.) chemically competent cells. Subsequent transformants were screened by colony PCR using primers supplied by the manufacturer: pRham Forward, 5′-GCTTTTTAGACTGGTCGTAGGGAG-3′ and pETite Reverse, 5′-CTCAAGACCCGTTTAGAGGC-3′. The sequence of two independent clones (pRHAM-LifA-6xH) were confirmed to be identical to the published *lifA* sequence of E2348/69 (gene E2348C_3234; (18)) by full-length Sanger sequencing on both strands using primers every 500 bp (GATC-Biotech).

##### Generation of a DTD to AAA Lymphostatin Substitution Mutant

To remove the D*X*D motif the amino acids DTD at position 557–559 were substituted with AAA using the QuikChange II XL site-directed mutagenesis kit (Agilent Technologies) according to the manufacturer's directions. Briefly, using the pRHAM-LifA-6xH plasmid as a template, the primers LifA-DXD-1 (Forward), GGATGTATATCCTTAAAGAGCATGGTGGTATTTATACA**GCGGCCGC**GATGATGCCTGCATACTCTAAACAAGTAATTTTTAAAA, and LifA-DXD-2 (Reverse), TTTTAAAAATTACTTGTTTAGAGTATGCAGGCATCAT**CGCGGCCG**CTGTATAAATACCACCATGCTCTTTAAGGATATACATCC, were used to introduce AAA to the sequence, by total replication of the plasmid, followed by digestion of the parent plasmid. A NotI restriction endonuclease site (indicated in bold) was also introduced at the site of mutation. Putative mutant plasmids were screened by restriction digest with NotI, and verified by Sanger sequencing on both strands (GATC-Biotech).

##### Recombinant Lymphostatin Expression and Purification

Recombinant His-tagged lymphostatin (rLifA) was overexpressed in *E. cloni*® cells cultured in lysogeny broth at 37 °C, 250 rpm shaking to *A*_600 nm_ 0.8. Expression of the protein was induced by the addition of l-rhamnose to 0.2% (w/v) and cultured for a further 3 h at 30 °C. Cells were pelleted by centrifugation, re-suspended in 20 mm sodium phosphate (NaH_2_PO_4_), pH 7.6, 300 mm sodium chloride, 500 mm non-detergent sulfobetaine (NDSB201), 20 mm imidazole, 5% (v/v) glycerol, 1 mm dithiothreitol (DTT), 100 μm phenylmethylsulfonyl fluoride, 1 complete protease inhibitor tablet/3 g cell mass (Roche), 0.1% (v/v) Tween 20 and lysed by high pressure lysis by single passage, at 30 kpsi, through a Constant Systems TS 1.1 kW Benchtop Cell disruptor. All chromatography was performed on an ÄKTA Explorer 10 UV900 LC system (GE Healthcare) at 6 °C. The lysate was clarified by centrifugation (50,000 × *g* at 4 °C) and loaded onto a Ni^2+^ ion-metal affinity chromatography (IMAC) column (HisTrap FF; GE Healthcare) pre-equilibrated in 20 mm sodium phosphate, pH 7.6, 300 mm sodium chloride, 20 mm imidazole, 5% (v/v) glycerol, 1 mm DTT, 0.1% (v/v) Tween 20 at 2 ml/min. This was followed by washing with 15 column volumes (cv) of the same buffer (Buffer A) and 15 cv of Buffer A supplemented with 4% Buffer B (20 mm sodium phosphate, pH 7.6, 300 mm sodium chloride, 500 mm imidazole, 5% (v/v) glycerol, 1 mm DTT, 0.1% (v/v) Tween 20). rLifA was eluted by increasing the concentration of imidazole up to 500 mm over 5 cv (gradient over 5 cv); all at 2 ml/min. rLifA was passed over a size exclusion column pre-equilibrated in 20 mm sodium phosphate, pH 7.6, 300 mm sodium chloride, 5% (v/v) glycerol, 1 mm DTT, 0.1% (v/v) Tween 20 to separate low molecular weight contaminants (Superose-6pg XK16/60; GE Healthcare) at 1 ml/min. Fractions containing rLifA were buffer exchanged into 15 mm sodium phosphate, pH 7.6, 50 mm sodium chloride, 5% (v/v) glycerol, 1 mm DTT, 0.05% (v/v) Tween 20 at 8 ml/min and further purified to homogeneity by anion exchange (HiPrep desalt 26/10; Mono-Q 5/50 GL; GE Healthcare) at 1 ml/min. The protein was eluted by running a gradient from 15 to 500 mm NaCl over 30 cv; the protein eluted at a salt concentration of 160 mm. AAA mutant full-length lymphostatin (rLifA^DTD/AAA^) was purified using the same strategy as rLifA protein, and the chromatograms were indistinguishable. The induction, size, and stability of expressed proteins were assessed by 3–8% Tris acetate SDS-PAGE and/or by Western blotting with monoclonal antibody specific for His_6_ (4A12E4, Novex, Life Technologies) according to the manufacturer's instructions (Novagen).

##### Isolation of Peripheral Blood Mononuclear Cells (PBMCs) and T Cells from Bovine Blood

Access to bovine blood for these studies was approved by the local ethics committee and blood draws were carried out in accordance with the Animals (Scientific Procedures) Act 1986. PBMCs were isolated from 12–18-month-old Holstein-Friesian cows. Briefly, blood was collected into heparinized bags or syringes. Following centrifugation at 1,200 × *g* for 15 min to generate an initial buffy coat, the white blood cell fractions were pooled, layered over Ficoll-Paque Plus (GE Healthcare), and centrifuged for 30 min at 1,200 × *g* with the brake off. The PBMCs were collected from the interface and washed several times before use. If required, the T lymphocyte fraction was further enriched using a sterile wool column (Polysciences, Inc.), as suggested by the manufacturer. Briefly, columns were washed in sterile Roswell Park Memorial Institute medium (RPMI) supplemented with 10% (v/v) fetal bovine serum, 20 mm Hepes, 1 mm sodium pyruvate, 100 units/ml of penicillin/streptomycin, 20 mm l-glutamine (Life Technologies), and incubated for 1 h at 37 °C in a 5% CO_2_ atmosphere. Cells were applied at 10^8^/ml, run into the column by gravity, and incubated for 1 h at 37 °C per 5% CO_2_. Unbound cells (mainly composed of T cells) were washed off the column in 10 ml of medium, spun down, and counted. The purity of T cell preparations was checked by single channel flow cytometry. Cells were stained with a commercially available anti-bovine CD3 antibody (MM1A; IgG1; VMRD, Pullman, WA). Secondary staining with a FITC-conjugated anti-IgG1 secondary antibody was carried out, and the samples were analyzed on a FACSCalibur using CellQuest (BD Biosciences) and FloJo software (Tree Star). A minimum of 10,000 events were collected, with an initial gate for live cells based on forward/side scatter parameters.

##### Proliferation Assay

Enriched T cells were used to test the activity of rLifA, purified partial fragments, and rLifA^DTD/AAA^ using a standard colorimetric measurement of mitogenic activation of proliferation. Cells were plated at 2 × 10^5^ cells/well in 96-well flat-bottom plates (Costar) in triplicate for all conditions. rLifA, rLifA^DTD/AAA^, or a fragment thereof was added at a final concentration as indicated in the figure legends. Cell proliferation was stimulated using the mitogen concanavalin A (ConA, Sigma) at a final concentration of 1 μg/ml in the presence or absence of recombinant lymphostatin as indicated in a final volume of 100 μl/well. Cells were incubated at 37 °C for 72 h. The colorimetric substrate CellTiter 96® AQueous One (Promega) was added 18 h before the end of the assay. All measurements were carried out at 492 nm on a Multiskan Ascent plate reader (Thermo Scientific). Cells and medium alone were used as negative controls. Background medium measurements were subtracted from all values. All treatments are expressed as a Proliferation Index, which is calculated by the ratio of: [absorbance (cells treated with ConA and recombinant protein)/absorbance (cells treated with ConA alone)]. Isolated T cells were tested for cytotoxic effect of rLifA using a lactate dehydrogenase release assay (Cytotoxicity Detection Test Plus, Roche) according to the manufacturer's directions, using an 8-h incubation period.

##### Limited Proteolysis and MALDI Mass Spectrometry

The identity of full-length recombinant lymphostatin was confirmed by in-gel protein digest and peptide analysis. Excised gel-bands were incubated at a porcine trypsin:lymphostatin ratio of ∼1:30, in 50 mm ammonium bicarbonate overnight at 32 °C (Promega). Peptides were identified by matrix-assisted laser desorption ionization (MALDI) mass spectroscopy on a Voyager DE-STR MALDI-TOF mass spectrometer (Applied Biosystems) using an α-cyano-4-hydroxycinnamic acid matrix. The spectral data were processed using Data Explorer software (Applied Biosystems) and the MASCOT NCBInr database searched against the peptide mass map (Matrix Science). To investigate the domain structure of lymphostatin, purified protein was incubated with trypsin at a ratio of 375:1, at 21 °C, to give limited digestion. Aliquots were removed at 1, 2, 3, and 4 h and the reaction stopped by boiling samples adjusted with 2 mm EDTA and 2 mm PMSF in SDS-PAGE loading buffer. Digest products were separated by SDS-PAGE and individual bands were subjected to in-gel tryptic digestion and MALDI-TOF mass spectroscopy as described above. Peptide masses were compared with the sequence of full-length rLifA using GPMAW 9.2 software, mass tolerance 50 ppm (19). Fragment F1 was purified to homogeneity from other digest products by ion-exchange chromatography (Mono-Q 5/50 GL; GE Healthcare) as described above.

##### Size Exclusion Chromatography-Multi-angled Light Scattering (SEC-MALS)

Size exclusion chromatography coupled to UV, static light scattering, and refractive index detection were used to determine the molecular mass of pure rLifA in solution and to estimate the detergent load (Viscotec SEC-MALS 20 and Viscotek RI Detector:VE3580; Malvern Instruments). One hundred μl of 1 mg/ml of lymphostatin was passed over a size exclusion column pre-equilibrated in 20 mm sodium phosphate, pH 7.6, 150 mm sodium chloride, 1 mm DTT, 0.1% Tween 20 (Superose6 10/300 GL, GE Healthcare) at 24 °C. Light scattering, refractive index (RI), and *A*_280 nm_ were analyzed by a conjugate protein model using the following parameters: *A*_280 nm_ for lymphostatin and Tween 20 detergent, 0.97 and 0.01 absorbance unit, ml/mg, respectively; RI for protein, 0.187 ml/g; and Tween 20, 0.145 ml/g (Malvern Instrument software).

##### Circular Dichroism (CD)

The far UV CD spectrum of full-length lymphostatin (0.11 μm), rLifA^DTD/AAA^ (0.11 μm), and the digest fragment F1 (0.35 μm) were recorded at 10 nm/min; data pitch, 0.1 nm; response time, 2 s between 185 and 285 nm in a 0.1-cm path length quartz cuvette at 25 °C (JASCO-810 spectrometer). The proteins were exchanged into 10 mm sodium phosphate, pH 7.6, 150 mm sodium fluoride prior to analysis (HiTrap desalt column, GE) at 4 ml/min. Spectra were corrected by subtracting a buffer baseline, each an average of 5 spectra. Secondary structure was estimated using the Dichroweb CD secondary structure analysis server (20) including the methods CONTIN, SELCON3, and CDSSTR (21–24) and reference data sets SP175 and 7 (25).

##### Bioinformatic Analysis

The secondary structure of lymphostatin was predicted from sequence using PredictProtein (26) and PSIPRED (27). Proteins with similar structural elements and homologues were identified with PHYRE (28) and BLASTp (29).

##### Intrinsic Tryptophan Fluorescence

Binding of uridine diphosphate-glucose (UDP-Glc) and UDP-GlcNAc to wild-type LifA and LifA^DTD/AAA^ was determined by ligand-induced changes in intrinsic tryptophan fluorescence. Fluorescence measurements were performed on a SPEX Fluoromax 3 spectrometer (Horiba) in a 3-ml stirred cuvette by titrating the UDP-sugar into 0.2 μm lymphostatin at 20 °C in 20 mm sodium phosphate, pH 7.6, 150 mm sodium chloride, 5% glycerol, 0.1% Tween 20, 1 mm DTT. Samples were allowed to equilibrate for 5 min after the addition of each aliquot. The final volume added did not exceed 2% of the initial volume. Tryptophan was excited at 295 nm and emission spectra were recorded from 310 to 400 nm, with a 1-nm interval. Excitation and emission slits were set at 5 nm, with an integration time of 1 s. The equilibrium dissociation constant was obtained from fitting the fluorescence intensity at 340 nm to a binding model corrected for collisional quenching (Kaleidagraph, Synergy Software).

##### Negative Staining and Electron Microscopy

Four hundred mesh carbon-coated copper grids were glow discharged for 1 min in a Quorum Tech sputter coater with a current of 25 μA and used within 1 h. rLifA (4 μl at ∼8 μg/ml concentration) was applied to a glow discharged grid and incubated for 1–2 min. Next, the grid was washed with 2 drops of water and 2 drops of 2% uranyl acetate followed by staining with a further drop of 2% uranyl acetate for 4–5 min. Finally, excess liquid was blotted from the edge of the grid with filter paper (Whatman No. 5). Dried grids were imaged with an FEI F20 field emission gun electron microscope equipped with an 8k x 8k TVIPS CMOS camera (F816). The camera format was binned by a factor of 2 giving a calibrated binned pixel size of 3.06 Å/pixel at the specimen level. Micrographs were acquired semi-automatically with EM-tools (TVIPS GmbH) under low dose conditions (20 *e*/Å^2^, at 200 kV).

##### EM Image Processing

The defocus of each micrograph was determined with ctffind3 (30). Particles were selected semi-automatically using e2boxer (31). Selected particle images were extracted and normalized with RELION (32), with a box size of 128 pixels. The radius for background normalization was 55 pixels. Extracted particle images were classified into 100 two-dimensional classes using RELION. Particle images, which did not align with an accuracy of better than 4 degrees in the two-dimensional classification were excluded from further processing. This reduced the number of particle images in the data set from 27,431 to 25,244. The relative spatial orientations of two-dimensional class averages of the remaining particle images were determined by sinogram correlation in IMAGIC (33). After determining the relative orientations of the class averages a three-dimensional map was calculated by weighted back projection. The orientations of the class averages were further refined by angular reconstitution using projections of the three-dimensional map as anchor set followed by calculating an improved three-dimensional map by back-projection. The resulting three-dimensional map was used as reference in Relion for autorefinement of the data set. The resolution was estimated by Fourier-Shell correlation between two independently processed data sets (34) and was 23 Å at a Fourier-Shell correlation of 0.14 (35). The absolute hand of the map is unknown.

##### Small-angle X-ray Scattering (SAXS)

Synchrotron radiation data were collected at the I911-4 SAXS beamline at the MAX-II Laboratory (Lund, Sweden) as 4 × 30-s exposures of a 20–30-μl sample and scattering profiles were compared with detect radiation damage. Lymphostatin was buffer exchanged into 20 mm NaH_2_PO_4_, pH 7.6, 300 mm NaCl_2_, 2 mm DTT, and 5% (v/v) glycerol (an identical buffer to the size-exclusion storage buffer but without Tween 20) prior to SAXS measurements using Amicon Ultra 0.5-ml centrifugal filters (Merck). Data were collected at 0.91-Å wavelength at 10 °C with a Dectis hybrid pixel Pilatus 1M detector. To detect concentration-dependent inter-particle effects, measurements were collected at multiple protein concentrations in the range of 0.67–1.6 mg/ml, and the 1 mg/ml scattering curve was used for all subsequent analysis. Background buffer scattering was subtracted using PRIMUS (36), part of the ATSAS package (37). Pair distance distribution function *P*(*r*) and the maximum particle dimension *D*_max_ were computed using GNOM (38). The Porod volume was calculated using ATSAS AUTOPOROD (39) and used for molecular weight estimation. *Ab initio* shape envelope was developed using 10 independent DAMMIF (40) computations in P1 symmetry. DAMAVER (41) was used to align and compare the resulting models. The most representative model was subsequently refined using DAMMIN (42). The resulting bead model was used to compute a surface envelope with Situs pdb2vol (43). The DAMMIN model was manually aligned with the EM density map using USCF Chimera (44).

##### Statistical Analysis of ED_50_ of rLifA and rLifA^DTD/AAA^

The effective dose 50 (ED_50_) for rLifA and rLifA^DTD/AAA^ was determined using drc in R (45). One way analysis of variance was used to determine statistically significant differences between both ED_50_ and dose-response curves using Minitab (46), with *p* values ≤0.05 taken to be significant. Post hoc Tukey test was used to calculate 95% confidence intervals and confirm significance (46).

## Results

### 

#### 

##### Full-length Lymphostatin Can Be Produced as a Stable, Tagged Recombinant Protein

**FIGURE 1.**
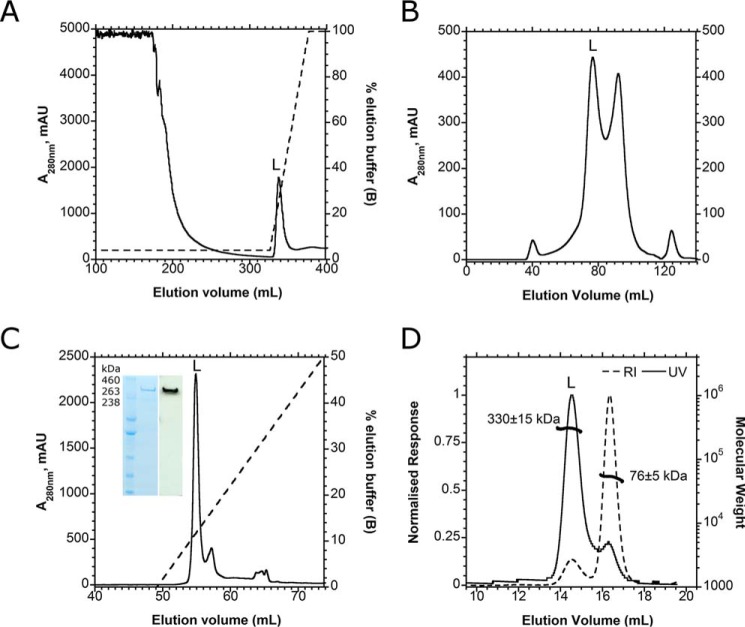
**Multiple-step purification of rLifA yields highly pure, full-length monomeric protein.** *A*, ion metal affinity chromatography (HiTrap IMAC FF) elution profile; lymphostatin (*L*) was eluted over a 4–100% gradient of Buffer B. *B*, size exclusion chromatography separated lymphostatin from lower molecular weight contaminants from the expression system (Superose 6pg XK16/60). *C*, ion exchange chromatography (Mono-Q 5/50 GL) was used as a final polishing step; full-length lymphostatin eluted at 160 mm NaCl. *Inset*, Coomassie-stained gel of 0.5 μg of purified full-length lymphostatin (*left lane*), Western blot of 0.1 μg of purified full-length lymphostatin probed with anti-His_6_ antibody (*right lane*). *D*, SEC-MALS chromatogram of purified lymphostatin (Superose 6 10/300 GL): *A*_280 nm_ (*solid line*) and refractive index trace (*dashed line*). Lymphostatin eluted at 14.5 ml and has an estimated mass of 330 ± 15 kDa; detergent micelle eluted separately with an estimated mass of 76 ± 5 kDa (*thick black trace*).

Published studies have noted instability of lymphostatin clones (2, 47) or inability to assemble the full-length gene from amplicons (8), possibly because of toxicity and gene size. To overcome this, an amplicon of the EPEC O127:H6 E2348/69 full-length *lifA* gene (9.6 kb) was cloned in pRham with a carboxyl-terminal histidine tag. In this Expresso Rhamnose cloning system, tight control of expression was achieved using a combination of glucose to repress transcription from the rhamnose-inducible promoter and a 3-h induction with 0.2% (v/v) l-rhamnose. Purification was optimized as described under “Experimental Procedures,” and Fig. 1, *A–C*, illustrates the steps in the purification strategy. No evidence of lymphostatin production was detected in uninduced cultures of *E. coli* harboring sequence-verified *lifA*, however, l-rhamnose induction led to the expression of rLifA, as detected by Coomassie staining and Western blotting with an anti-His tag antibody (Fig. 1*C*, *inset*).

To confirm that the expressed protein is full-length lymphostatin, in-gel tryptic digestion and MALDI mass spectrometry were carried out. The resulting peptides aligned to 41% of the lymphostatin primary sequence, from residue 52 to 3174 (of 3229); representing good coverage for such a large protein. The predicted N terminus of the protein is relatively rich in basic residues. Lack of tryptic peptide coverage at the N terminus could be accounted for by extensive digestion at charged residues generating many low mass fragments that are not detectable by MALDI. The C-terminal end can be inferred to be intact because of detection of the histidine tag and by IMAC purification and Western blotting. The purified protein was analyzed by dynamic light scattering. The sample contained species with a Stokes radius of 6.5 nm, consistent with monomeric rLifA. There was no evidence of aggregation or insolubility (data not shown).

##### rLifA Is Biologically Active against Bovine T Cells

**FIGURE 2.**
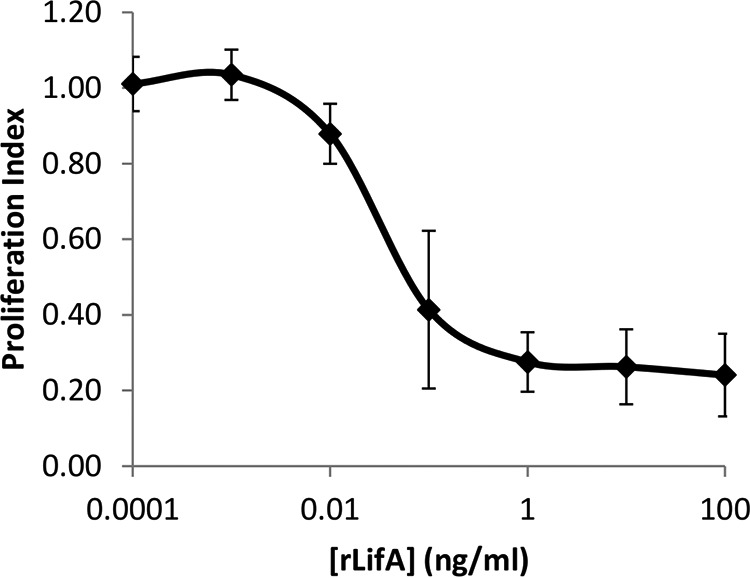
**Concentration-dependent inhibition of T cell proliferation by full-length rLifA.** Effect of concentration of lymphostatin against ConA-stimulated peripheral bovine T cells. Data shown are the average of 4 independent donors, done in triplicate. *Error bars* indicate S.E. The purity of the cells used was >85% in each experiment, as measured by CD3 staining using flow cytometry. Calculated ED_50_ was 25 ± 4.6 pg/ml (68 fm); 100 ng/ml is equivalent to ∼273 pm. Concentrations on the *x* axis are displayed on a log scale.

Lymphostatin-containing bacterial lysates have been reported to be capable of inhibiting the proliferation of mitogen-stimulated PBMCs using cells from humans, mice (2, 7), and cattle (3, 11). Using an enriched bovine T cell population increased the signal-to-noise ratio compared with use of bulk PBMCs, giving a wider dynamic range to the assay. Using T cells obtained from four independent donors, purified rLifA inhibited ConA-stimulated proliferation, showing inhibition from the low femtomolar range with concentration-dependent titration of activity with a sigmoidal curve (Fig. 2). Measurements of the ConA-stimulated response were typically 2–5-fold higher than cells alone. The carrier buffer for rLifA was determined to have no effect on ConA stimulation of cells on its own. Furthermore, a colorimetric assay to detect release of cytosolic lactate dehydrogenase found no evidence of cell lysis when primary T cells were treated with inhibitory concentrations of rLifA, indicating that the inhibitory effect of lymphostatin on T cells is unlikely to be a consequence of direct cytotoxicity.

##### Lymphostatin Is a Monomer in Solution

Having established that rLifA is biologically active, we examined its structural characteristics. SEC-MALS was used to calculate the average molecular weight of rLifA in solution and hence the oligomeric assembly. Lymphostatin eluted as a well resolved single peak. Light scattering, RI, and UV were analyzed by a conjugate protein model, and the mass of lymphostatin was predicted to be 330 ± 15 kDa, slightly lower than the predicted molecular mass for monomeric protein of 365 kDa (Fig. 1*D*). Given the mapping of the N-terminal to at least 52 residues from the beginning of the putative start of the protein, and that the C-terminal is intact, the size differential to the calculated size is unlikely to be due to truncated protein, and is within acceptable error for the technique at the present time, taking into account the glycerol and detergent-rich buffer. Detergent micelle eluted later than protein with an estimated mass of 76 ± 5 kDa (Fig. 1*D*). Taken together the data indicate that lymphostatin is a monomer in solution and that it is not necessary for the protein to fully or partially be enclosed within a micelle to remain in solution.

##### Limited Proteolysis Identified 3 Putative Structural Domains

**FIGURE 3.**
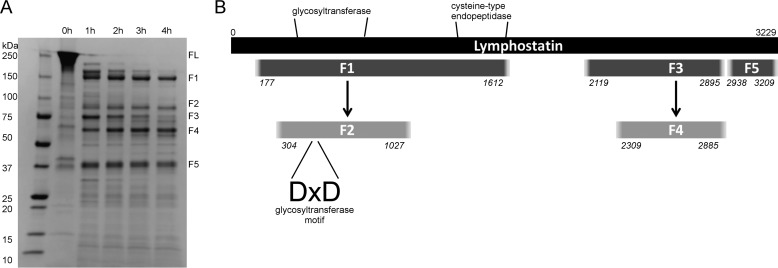
**Domain organization of lymphostatin.** *A*, limited proteolysis of lymphostatin gave a consistent digest pattern. Five fragments, *F1–F5*, were identified by mass spectrometry. *B*, analysis of the primary amino acid sequence of lymphostatin predicts a glycosyltransferase (*GT*) and cysteine-type endopeptidase domain (*CP*). *Black bar* illustrates full-length protein (*FL*). Fragment F1 contains the putative GT and CP domains. Fragment F2 maps more closely to the GT domain with a C-terminal extension of ∼140 aa.

With no fine structural information available on lymphostatin we set out to predict putative physical domains using limited proteolysis; the premise being that loops and flexible regions of the protein are more susceptible to digestion than buried residues. Limited tryptic proteolysis of rLifA over several hours revealed a defined and consistent pattern of cleavage products. Five major fragments were identified by mass spectrometry, F1-F5 (Fig. 3*A*). Mass spectrometry of tryptic peptides from each species and alignment of peptides against/with the lymphostatin primary sequence suggested 3 major domains, with the additional two species representing truncated versions of two of the major digestion products, as indicated in Fig. 3*B*. Identification of peptides by MALDI-TOF mass spectrometry from the in-gel tryptic digestion of fragments initially generated by limited proteolysis does not precisely identify protein domain boundaries but may be considered a useful guide. Limited proteolysis fragments are likely to be somewhat longer at both the N and C terminus than the most N-terminal and C-terminal residues identified from their tryptic digestion and MS analysis. The largest intact fragment, designated F1, represents a ∼1435 amino acid (aa) region at the N terminus of lymphostatin, starting about 177 aa from the N terminus, and encompassing both the putative glycosyltransferase (GT) and cysteine protease (CP) motifs. The two smaller fragments are separated from the N-terminal fragment by about 500 aa, and are approximately ∼776 aa (F3) and 271 aa (F5), respectively. In addition, the two C-terminal proximal fragments are separated by a short stretch of 43 aa. Residues 1600–2100 are not represented in the major early digest products but were very well represented in the full tryptic digest during mass spectrometry analysis, suggesting this region is intact in the full-length protein. The abundance of fragment F1 reduces as the digest time increases, whereas fragment F2, an N and C terminally truncated subfragment of F1, becomes relatively more abundant. Fragment F2 maps more closely to the GT domain with a C-terminal extension of ∼140 aa. The N-terminal ∼100 aa of F1 is richer in basic amino acids than the full-length lymphostatin. This property enabled us to separate F1 from other digest products by anion exchange chromatography.

##### The Predicted Glycosyltransferase Domain of Lymphostatin

**FIGURE 4.**
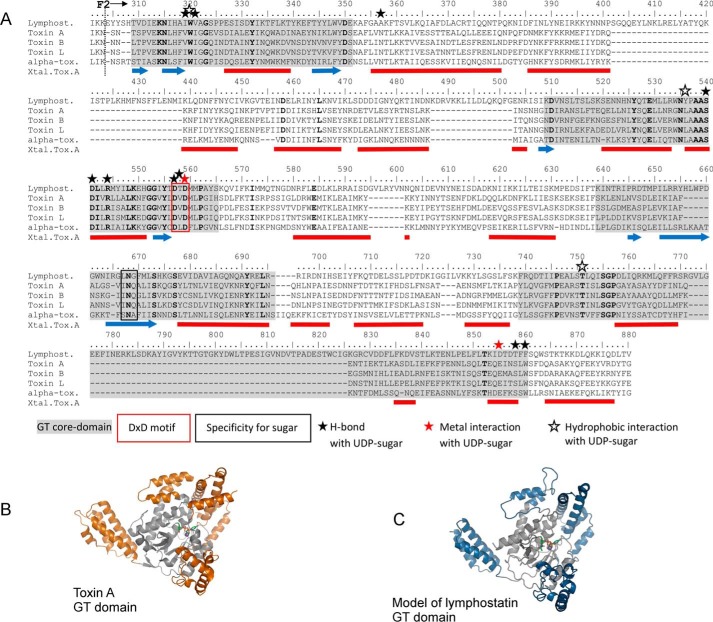
**The putative glycosyltransferase domain of lymphostatin.** *A*, alignment of sequence representing the putative GT domain of lymphostatin with those of Toxin A and B from *C. difficile*, Toxin L from *Clostridium sordellii*, and α-toxin from *Clostridium novyi*. Sequences were aligned using the program Clustal Omega (66). *Red bars* represent α-helical regions and *blue arrows* represent β-sheet from the crystallographic analysis of Toxin A from *C. difficile* (Protein Data Bank code 4DMW (53)). Amino acid identities between the LCTs and lymphostatin are indicated in *bold*. Residues that make key interactions with the UDP-sugar in the LCTs are indicated with *stars*. The core catalytic fold that defines the UDP-Glc donor substrate binding site of the LCTs is indicated with *gray shading* in both the sequence alignment and structural representations. The numbering scheme refers to lymphostatin. *F2* indicated the N-terminal boundary of digest fragment F2. *B*, representation of the crystal structure of toxin A from *C. difficile* bound to UDP-Glc (Protein Data Bank code 4DMW) (67). *C*, PHYRE2 (49) generated model of the GT domain of lymphostatin based on Toxin A from *C. difficile*. UDP-Glc extracted from Protein Data Bank code 4DMW was modified to UDP-GlcNac and fitted into the putative binding site using C*oot* (48).

**FIGURE 5.**
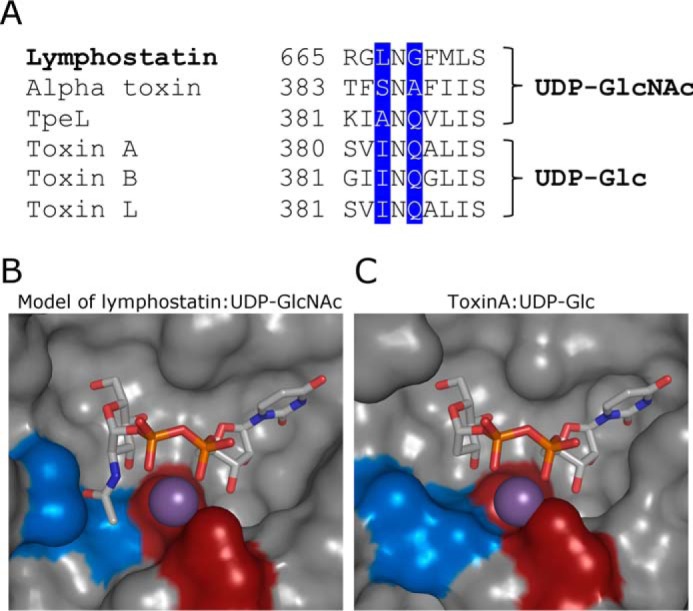
**Modeling donor substrate specificity of lymphostatin by comparison to clostridial toxins.** *A*, alignment of the sequence of lymphostatin with those LCT residues thought to contribute to specificity for UDP-sugar. *B*, PHYRE2 (49) generated model of the GT domain of lymphostatin in complex with UDP-GlcNac based on Toxin A from *C. difficile*. Leu^667^ and Gly^669^ are shown in *blue* and Asp^557^ and Asp^559^ in *red. C*, toxin A from *C. difficile* in complex with UDP-Glc. Ile^382^ and Gln^384^ are shown in *blue* and Asp^285^ and Asp^287^ in *red*.

To identify the boundaries of the glycosyltransferase domain of lymphostatin we performed BLAST analysis of the primary sequence. We identified homology with the N termini of LCTs (the first 541 amino acids of TcdA) encompassing their catalytic glycosyltransferase domains. The alignment indicates that the sequence of lymphostatin is most similar to that of the LCTs for residues that define the binding site of the glycosyl donor substrate, often described at the catalytic core of the GT domain (Fig. 4*A*, *gray shading*) (13). The catalytic core residues of the LCTs are not contiguous and have insertions that form helical bundles surrounding the core (Fig. 4*B*, Toxin A, *orange highlights*). These are thought to confer specificity for the protein that becomes glycosylated by the glycosyltransferase. The most striking differences in sequences in the LCT GT domains are within these insertions. To further examine the potential of lymphostatin to form a GT fold we generated a model using the PHYRE2 server (49). The best scoring model is based on Toxin A from *Clostridium difficile*, which forms a GT-A-fold common to all LCTs (Fig. 4*C*; model of lymphostatin Ala^242^-Arg^769^). Lymphostatin has similar insertions that are predicted from the sequence to be coiled and helical in character. It is worth noting that differences to the helical bundles mean that even if an alignment is restricted to the catalytic core of the clostridial toxins (residues Thr^105^-Trp^534^ in TcdA, which align to residues Thr^309^-Phe^860^ in lymphostatin) the sequence identity is only 20%, a figure that belies their common features. Lymphostatin displays good conservation for residues that make key non-covalent interactions with the UDP-sugar (Fig. 4*A*, *stars above* sequence; *bold typeface* marks identity). The LCTs and lymphostatin contain the signature D*X*D motif, seen in most GT domains, through which the aspartate carboxylates coordinate a divalent cation and the donor substrate (Fig. 4*A*, *red box*). Three residues at the base of the β-hairpin in the sugar donor binding site, *X*N*X*, are thought to confer sugar specificity (Figs. 4*A*, *black box*, and 5, *A* and *C*). Toxins A, B, and L all have INQ in this position; this means that the enzymes can accommodate UDP-Glc but not UDP-GlcNAc. α-Toxin and TpeL bind UDP-GlcNac; to accommodate the acetyl group, INQ is replaced by SNA and ANQ, respectively. Lymphostatin has leucine in position 1 and glycine in position 3, LNG. Our model suggests this would make it possible for UDP-GlcNAc to bind (Fig. 5*B*).

**FIGURE 6.**
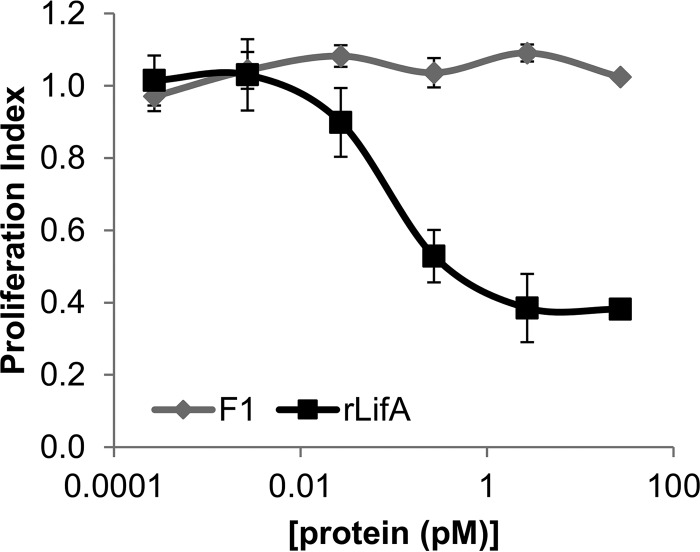
**The putative glycosyltransferase domain of lymphostatin is not sufficient in isolation to inhibit T cell proliferation.** Effect of concentration of rLifA and the purified partial tryptic digestion fragment F1 against ConA-stimulated peripheral bovine T cells. Data shown are the average of 4 independent donors, done in triplicate. *Error bars* indicate the S.E. The purity of the input cells used was >85% in each experiment, as measured by CD3 staining using flow cytometry. Concentrations on the *x* axis are displayed on a log scale.

Given that digest fragment F1 spans both the putative glycosyltransferase motif as well as the cysteine protease motif; we set out to test the F1 fragment in isolation in the bovine T cell proliferation assay, to explore whether it retained its inhibitory activity against lymphocytes. Using equimolar amounts of protein in the ConA-stimulated T cell proliferation assay, comparison of the full-length protein alongside purified F1 revealed that the F1 fragment was insufficient to inhibit T cell proliferation in isolation (Fig. 6). This may be because the F1 fragment N-terminal boundary is at ∼177 aa and the N-terminal amino acids are important for interaction with the target protein. More likely is that the C-terminal domains are crucial for cell binding and uptake, as has been shown for the large clostridial toxins (50).

##### Lymphostatin Is an Ordered Protein with a High α-Helical Content in the Putative Glycosyltransferase Domain

**FIGURE 7.**
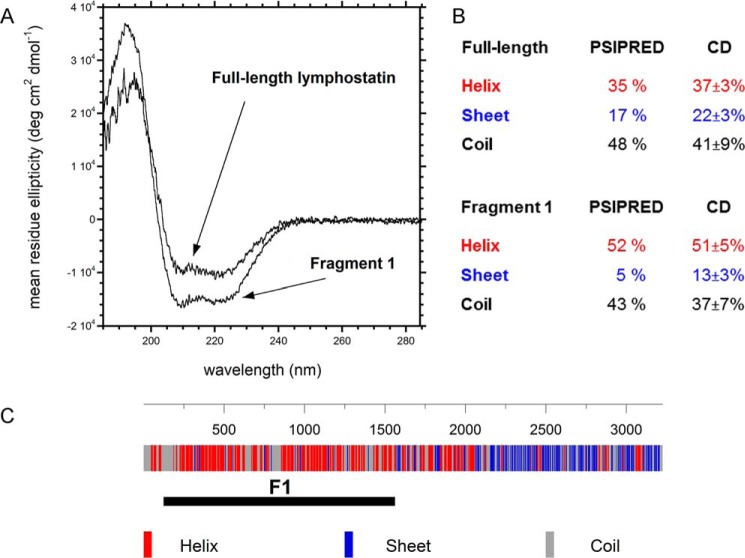
**Secondary structure analysis.** *A*, far UV CD spectrum of lymphostatin and the major N-terminal fragment F1. *B*, secondary structure analysis from CD and predictions from primary sequence indicate that F1 is largely α-helical. There is good agreement between PSIPRED predictions and secondary structure analysis of the CD data for full-length lymphostatin. *C*, secondary structure prediction from primary sequence obtained from PSIPRED.

To confirm that rLifA was folded and to estimate the content of secondary structural elements we measured the CD spectrum of full-length and the F1 fragment that encompasses the putative GT and the CP domains. The CD spectrum of full-length protein was consistent with a folded protein and indicated 37% α-helix and 17% β-sheet (Fig. 7, *A* and *B*); very similar to PSIPRED predictions (∼35% α-helical and ∼22% β-sheet). The N-terminal half of the protein was predicted to contain the majority of the α-helical content, whereas the C-terminal third of the protein was predicted to be rich in β-sheet (Fig. 7*C*). The samples showed no evidence of aggregation by dynamic light scattering (ZetasizerAPS, Malvern).

Given the lack of activity of the isolated F1 fragment in the T cell proliferation assay, and to rule out that the protein was degraded and/or not folded, CD analysis was carried out on purified F1 protein. Furthermore, given its location at the N-terminal part of lymphostatin, where the primary sequence is predicted to have a higher percentage of α-helices, it is expected that the percentage of α-helical content of F1 would be higher than that of the full-length protein. The F1 fragment was predicted by PSIPRED to consist of 52% α-helical content and analysis of CD data inferred 51% α-helical content. The CD data is broadly in agreement with the secondary structure prediction for F1 but suggested a slightly higher proportion of β-sheet, 13% as opposed to 5%.

##### Three-dimensional Shape of Lymphostatin

**FIGURE 8.**
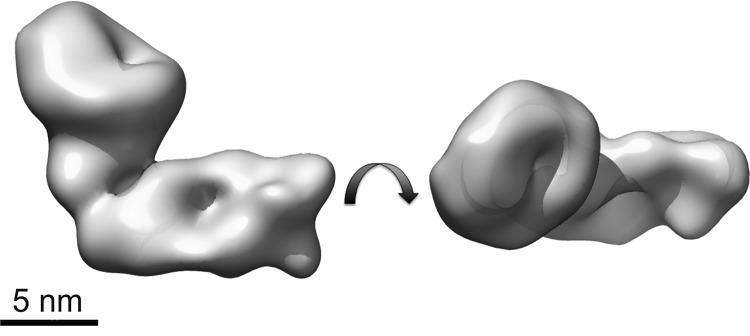
**Surface representation of negatively stained rLifA at 23-Å resolution.** The views are related by a 90 degree rotation around the horizontal axis. The *length of the scale bar* equals 5 nm.

**FIGURE 9.**
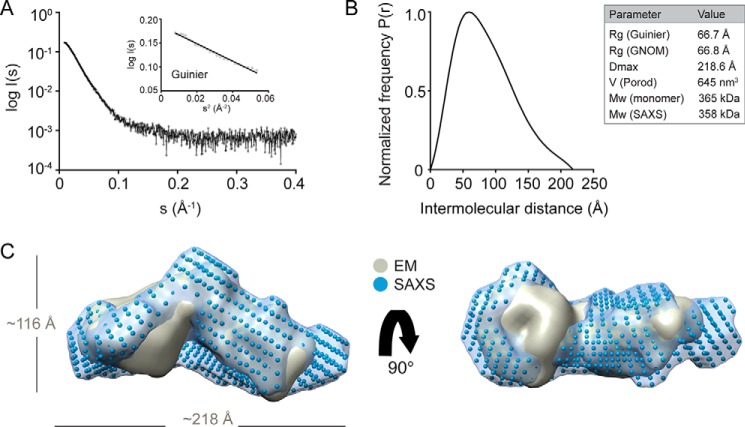
**SAXS analysis of rLifA.** *A*, the experimental SAXS scattering curve is shown as a logarithmic intensity I (s) *versus* inverse scattering angle (Å^−1^). The linear region of the Guinier plot (log *I*(s) *versus* s^2^) is shown as an *inset. B*, the distance distribution function *P*(*r*) with SAXS parameters shown in the *inset. R_g_*, radius of gyration; the *Mw* (*monomer*) is the mass calculated from the primary sequence; *Mw* (*SAXS*) is the estimated mass calculated from the Porod volume. *C*, the refined DAMMIN *ab initio* bead model with indicated dimensions (*blue*) and its *ab initio* surface envelope (*blue*) aligned with the EM density map (*gray*), shown in two views related by a 90 degree rotation around the horizontal axis. The SAXS envelope was made transparent with Chimera (44) to visualize the alignment.

Negatively stained rLifA showed a homogeneous distribution of elongated, slightly kinked particles (Fig. 8). From images of ∼25,000 of these particles, we determined a three-dimensional map of lymphostatin at 23-Å resolution. The map shows an elongated, L-shaped molecule (Fig. 8). The arms of the “L” are 130–140 Å long and between 60 and 90 Å thick. To further investigate the structure of lymphostatin, we also performed SAXS (51, 52). The linearity of the Guinier region confirms the absence of inter-particle effects (Fig. 9*A*). The longest particle dimension *D*_max_ is 218 Å, which is not dissimilar to the longest dimension of the EM map (∼197 Å) (Fig. 9*B*). The Porod volume (645 nm^3^) indicates a particle molecular mass of 358 kDa further suggesting that lymphostatin (365 kDa based on primary sequence) behaves as a monomer in solution, and that the recombinantly purified full-length protein is stable. Next, we determined the *ab initio* shape analysis with DAMMIF (40) using 10 independent computations producing models related with an average normalized spatial discrepancy of 0.793. The representative and DAMMIN (42) refined model shows an elongated L-shape with dimensions (218 × 116 × 100 Å) slightly larger than the EM density map (197 × 110 × 89 Å). The alignment of the EM density map with the SAXS envelope highlights the similarities in dimensions as well as shape (Fig. 9*C*). The kink of the L-shaped molecule is situated in a similar position in the two envelopes, at roughly half the length of the particle, however, the kink is more defined in the EM map. In addition, the EM map contains a larger volume within one of the arms of the L.

##### Lymphostatin Binds UDP-N-acetylglucosamine

**FIGURE 10.**
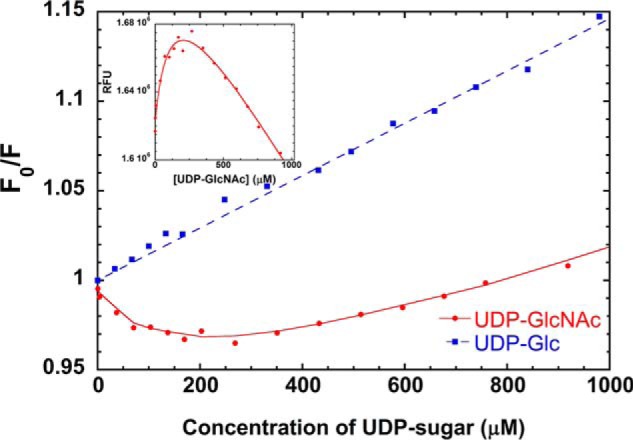
**Lymphostatin binds UDP-*N*-acetylglucosamine.** Stern-Volmer plot showing the intrinsic tryptophan fluorescence (340 nm) of lymphostatin, measured at increasing concentrations of UDP-*N*-acetyl-glucosamine (UDP-GlcNAc) and UDP-glucose (UDP-Glc) (*F_o_*/*F*; where *F_o_* is the fluorescent intensity before the addition of UDP-sugar). Data points represent the means of data in triplicate. *K_d_* for UDP-GlcNAc was determined to be 120 ± 30 μm by fitting the raw fluorescent intensity data to a one-site binding model corrected for collisional quenching (*inset*).

Lymphostatin contains 37 tryptophan residues, of which 7 are found within the putative GT domain; Trp^320^, Trp^534^, and Trp^863^ most likely are in close proximity to the active site (Fig. 4). Titration of lymphostatin with UDP-Glc gave a linear Stern-Volmer plot that would be consistent with collisional quenching of solvent-exposed tryptophan rather than any specific interaction of sugar with lymphostatin (Fig. 10). In contrast UDP-GlcNAc enhances fluorescence in a non-linear concentration-dependent manner at lower concentrations; there is evidence of collisional quenching at higher concentrations. This may be explained by UDP-GlcNAc binding in the GT catalytic site and changing the environment of one or more tryptophan residues. At high concentrations of UDP-GlcNAc after the active site has become saturated there is a linear relationship between fluorescence and UDP-GlcNAc concentration that could be attributed to collisional quenching of tryptophan outside the binding site. We have fitted a mixed binding model to the UDP-GlcNAc data that takes into account specific binding and collisional quenching. The affinity of lymphostatin for UDP-GlcNAc is estimated to be 120 ± 30 μm. The protein was expressed in the presence of a variety of divalent cations including Ca^2+^, Mg^2+^, and Mn^2+^ that were present in the growth media. Good reproducibility of affinity data between batches was achieved by incubating the protein sample with 50 μm MnCl_2_ after purification. Manganese was selected as the divalent cation based on thermal shift experiments (differential scanning fluorimetry) that suggested MnCl_2_ enhanced the thermal stability of lymphostatin and the additional stabilizing effect of UDP-GlcNac to a greater extent than MgCl_2_ (data not presented) (53). The *K_m_* of full-length TcdA from *C. difficile* for UDP-Glc in the presence of Mg^2+^ is 142 μm and has a *K_d_* of 45 ± 10 μm (54). The *K_m_* for the TcdA-GT domain has been reported as 36.3 ± 3.6 μm and *K_d_* of 11.4 ± 0.9 μm (53). *K_m_* of full-length TcdB from *C. difficile* for UDP-Glc in the presence of Mg^2+^ is 154 μm (55).

##### The DTD Motif within the GT Domain of Lymphostatin Is Important in Sugar Binding and Activity against Bovine Lymphocytes

Given that the D*X*D motif has been implicated in glycosyltransferase activity in other similar molecules (reviewed in Ref. 13), we examined the effect of a DTD to AAA substitution at position 557–559 on both sugar binding and activity of lymphostatin. rLifA^DTD/AAA^ behaved similarly to rLifA, both during expression and purification, as well as in CD and DLS lending confidence that the residue substitutions had not disrupted the original structure of the protein.

**FIGURE 11.**
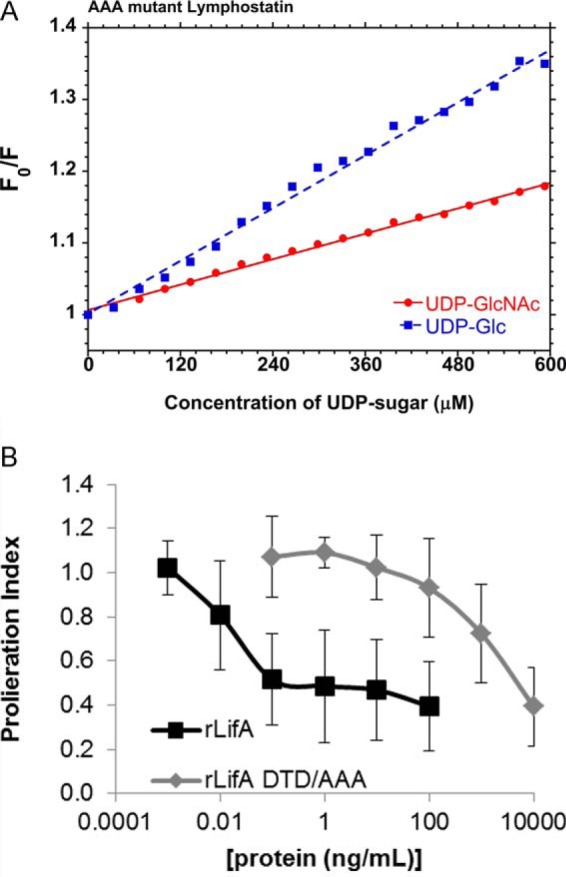
**Mutation of the D*X*D motif of lymphostatin abolishes both sugar binding and inhibitory activity.** *A*, Stern-Volmer plot showing the quenching of intrinsic lymphostatin tryptophan fluorescence (340 nm) measured at increasing concentrations of UDP-GlcNAc and UDP-Glc (*F_o_*/*F*; where *F_o_* is the fluorescent intensity before the addition of UDP-sugar). *B*, effect of concentration of rLifA and rLifA^DTD/AAA^ against ConA-stimulated proliferation of peripheral bovine T cells. The data shown are the average of 3 independent donors, done in triplicate. *Error bars* indicate S.E. The calculated ED_50_ was 0.014 ± 0.0015 ng/ml (rLifA), and 922 ± 270 ng/ml (rLIfA^DTD/AAA^). Concentrations on the *x* axis are displayed on a log scale.

Testing rLifA^DTD/AAA^ using the tryptophan fluorescence assay and titrating either UDP-Glc or UDP-GlcNAc as for rLifA gave linear Stern-Volmer plots indicative of nonspecific quenching and lack of sugar binding (Fig. 11*A*). Furthermore, titration of the rLifA^DTD/AAA^ protein in the bovine T cell proliferation assay shows a drastic reduction in its ability to inhibit proliferation by almost 4 orders of magnitude (ED_50_ rLifA = 0.014 ng/ml ± 0.0015, rLifA^DTD/AAA^ = 922 ng/ml ± 270) (Fig. 11*B*). These two observations imply that not only is the DTD motif important for sugar binding, but is also critical for the inhibitory activity of lymphostatin on T cells.

## Discussion

Lymphostatin plays an important role in intestinal colonization by attaching and effacing *E. coli* and is therefore a potentially attractive target for intervention. Although functions have been assigned to this large molecule, its mode of action remains largely unknown. It is one of relatively few bacterial molecules reported to interfere with the function of adaptive immune cells. Historically, it has proven difficult to obtain a stable clone to express and purify full-length lymphostatin (2, 8, 47). Using a tightly inducible prokaryotic expression system we were able to express and purify lymphostatin with potent activity against mitogen-stimulated T lymphocytes. Mass spectrometry of tryptic peptides and SAXS indicate that the purified full-length protein is stable. Lymphostatin contains predicted non-canonical prepilin peptidase sites (8), however, it is not clear whether it is processed, at least in the laboratory-adapted *E. coli* K-12 strain used for expression, nor is there a predicted signal sequence at the N terminus.

Since its discovery, two predicted features have been known in lymphostatin: namely a glycosyltransferase domain and a YopT-like cysteine protease domain (8, 17). Although both motifs were implicated in colonization of mice and colonic hyperplasia by *C. rodentium* (5), the mutations caused truncation of lymphostatin and subsequent studies with in-frame deletions suggested the motifs were not essential for intestinal colonization of cattle by enterohemeorrhagic *E. coli* O26:H− (4). However, the effect of lymphostatin and motif mutations on mucosal lymphocytes and induction of adaptive immunity was not studied and a role for the motifs in pathogenesis therefore cannot be excluded. Using purified lymphostatin we have not observed autocatalytic cleavage via the putative cysteine protease domain, including under low pH conditions or in the presence of inositol hexakisphosphate, the co-factor known to trigger autocatalytic cleavage of LCTs (reviewed in Ref. 56). If lymphostatin is able to autocatalytically cleave using the CP motif, either it requires some as yet unidentified co-factor, or it is not triggered by pH change, and merits further closer investigation.

Although we have obtained low resolution EM and SAXS envelopes for lymphostatin, a high resolution structure has been elusive. A full-length diffracting protein crystal has not yet been obtained from pilot crystallization trials. Given the behavior of the recombinant protein in solution, high resolution cryo-EM might be a promising approach to analyze the lymphostatin structure. Alternatively, given the data from the partial tryptic digestion, an approach combining crystallization of predicted domains combined with EM could be used. Circular dichroism spectroscopy of full-length and the N-terminal putative glycosyltransferase domain has confirmed the secondary structure predictions that show the N-terminal third of the protein to be largely α-helical. In addition, the C-terminal third is very rich in β-sheet, both features shared with the LCTs. Interestingly, unlike the LCTs, lymphostatin lacks the multimodular cell wall/choline-binding repeat that form the receptor-binding domain (57).

For the first time we report that lymphostatin is able to bind UDP-GlcNAc, but not UDP-Glc in a manner dependent on a conserved D*X*D motif within its predicted glycosyltransferase domain and that this motif is also required for its activity against bovine T lymphocytes. Furthermore, inhibition of lymphocyte function was not associated with cell lysis as measured by release of a cytosolic enzyme or effects on the actin cytoskeleton, in contrast to LCTs (data not shown) (58, 59). The target of sugar modification by lymphostatin is unknown, and is likely to be difficult to identify. For both the LCTs and NleB, there was some initial insight into rational candidate acceptor proteins or signaling pathways prior to their identification. However, this is not currently the case for lymphostatin. Furthermore, whereas potential targets of NleB activity were identified by immunoprecipitation and yeast 2-hybrid approaches, enabling specific sugar transfer onto putative recombinant targets to be tested (14, 15), this approach is likely to be technically challenging for lymphostatin, because of the challenges of cloning the gene in the vectors required for yeast 2-hybrid screening. Interacting partners for lymphostatin are presently unknown and a similar approach may be needed to find its target(s), given the challenge of detecting addition of labeled GlcNAc to cellular proteins against the large number of endogenous modifications expected.

Although lymphostatin has recently been demonstrated to be secreted via Type III secretion (10), the fact that it is active in an isolated recombinant form, or when produced in *E. coli* that lack a type III secretion system, indicates that injection into cells is not vital for activity against lymphocytes. It is plausible that it may have both Type III secretion-dependent and -independent activities. Our evidence indicates that the full-length protein is required for inhibition of lymphocyte function as the isolated F1 fragment encompassing the glycosyltransferase domain showed no activity, despite being folded and soluble. As with large clostridial toxins the C-terminal domain of lymphostatin may be required for cellular uptake and the GT domain is unable to act in isolation (60, 61).

Where EPEC and non-O157 EHEC almost invariably express lymphostatin, in serogroup O157 EHEC strains, lymphostatin exists as two truncated open reading frames (z4332 and z4333, encoding proteins identical to residues 1–433 and 435–710 of full-length lymphostatin) (62, 63). In addition, the pO157 plasmid encodes a putative 365-kDa homologue of lymphostatin named ToxB (L7095), which shares 28% identity and 47% amino acid similarity to LifA/Efa1 (64, 65). *E. coli* O157 strains have a lymphostatin-like activity (3, 15) that has been associated with the pO157 plasmid (2). Mutation of the truncated lymphostatin or *toxB* genes in a Shiga toxin-deficient *E. coli* O157 strain did not markedly affect the ability of bacterial lysates to inhibit bovine lymphocyte proliferation (11). However, the assay used relied on crude bacterial lysates and is insensitive compared with the highly purified protein tested herein, with lysates causing lymphostatin-independent inhibition of lymphocyte function at higher concentrations (4, 11). The expression and assay systems described here are likely to be suitable to assign activities to ToxB and truncated lymphostatin, and indeed a family of homologous cytotoxins described in pathogenic *Chlamydia* species (16). Almost two decades after lymphostatin was originally identified we have demonstrated that it has sugar-binding potential, that it is a highly potent inhibitor of T cell proliferation *in vitro*, and that a D*X*D motif predictive of glycosyltransferase function is essential for both these activities.

## Author Contributions

R. L. C. C., E. A. B., M. W., R. J., and M. P. S. contributed to conception and design of study; R. L. C. C. cloned the original expression construct and initial protein studied, carried out the T cell proliferation assays for full-length lymphostatin and the F1 fragment, analyzed data, constructed figures, and carried out statistical analysis, wrote the first draft of the manuscript, and coordinated revision and submission; E. A. B. optimized protein production and purification, carried out biophysical characterization and analysis, domain bioinformatic analysis, all CD analysis, sugar binding assays, and contributed to writing the first draft of the manuscript; H. A. carried out sugar binding assays and SAXS analysis for full-length lymphostatin; E. D. carried out SAXS analysis for full-length lymphostatin and contributed to writing the first draft of the manuscript; A. B. prepared the AAA mutant of lymphostatin, helped with optimization of production and purification, carried out proliferation and sugar binding assays using the mutant protein; B. B. carried out the EM staining, image acquisition and analysis to produce the three-dimensional reconstruction of lymphostatin, and contributed to writing the first draft of the manuscript; R. J. carried out SAXS analysis and contributed to writing the manuscript; M. P. S. contributed to writing the manuscript. All authors contributed to analyzing the data, editing the manuscript, and approved the final form.

## Footnotes

4 The abbreviations used are:

EHEC: enterohemorrhagic *E. coli*
EPEC: enteropathogenic *E. coli*
aa: amino acid
ConA: concanavalin A
CP: cysteine protease
cv: column volume
EHEC: factor for adherence 1
GT: glycosyl transferase
IMAC: ion-metal affinity column
LCT: large clostridial toxin
LifA: lymphocyte inhibiting factor A
RI: refractive index
SAXS: small-angle x-ray scattering
SEC-MALS: size exclusion chromatography multi-angled light scattering
UDP-Glc: uridine diphosphate glucose
UDP-GlcNAc: uridine diphosphate *N*-acetylglucosamine
PBMC: peripheral blood mononuclear cell.
